# OXA-48 Carbapenemase-Producing Enterobacterales in Spanish Hospitals: An Updated Comprehensive Review on a Rising Antimicrobial Resistance

**DOI:** 10.3390/antibiotics10010089

**Published:** 2021-01-18

**Authors:** Mario Rivera-Izquierdo, Antonio Jesús Láinez-Ramos-Bossini, Carlos Rivera-Izquierdo, Jairo López-Gómez, Nicolás Francisco Fernández-Martínez, Pablo Redruello-Guerrero, Luis Miguel Martín-delosReyes, Virginia Martínez-Ruiz, Elena Moreno-Roldán, Eladio Jiménez-Mejías

**Affiliations:** 1 Department of Preventive Medicine and Public Health, University of Granada, 18016 Granada, Spain; carlos.rivera.izquierdo.sspa@juntadeandalucia.es (C.R.-I.); luismiguelmr@ugr.es (L.M.M.-d.); virmruiz@ugr.es (V.M.-R.); elmorol@ugr.es (E.M.-R.); eladiojimenez@ugr.es (E.J.-M.); 2 Service of Preventive Medicine and Public Health, Hospital Clínico San Cecilio, 18016 Granada, Spain; 3 Biosanitary Institute of Granada, ibs.GRANADA, 18012 Granada, Spain; 4 Department of Radiology, University of Granada, 18016 Granada, Spain; ajbossini@ugr.es; 5 Service of Ginecology and Obstetrics, Hospital Universitario Virgen de las Nieves, 18014 Granada, Spain; 6 Service of Internal Medicine, San Cecilio University Hospital, 18016 Granada, Spain; jairo.lopez.sspa@juntadeandalucia.es; 7 Department of Preventive Medicine and Public Health, Reina Sofía University Hospital, 14004 Córdoba, Spain; nicolasf.fernandez.sspa@juntadeandalucia.es; 8 Maimonides Biomedical Research Institute of Córdoba (IMIBIC), 14001 Córdoba, Spain; 9 School of Medicine, University of Granada, 18016 Granada, Spain; pablorg239@correo.ugr.es; 10 CIBER of Epidemiology and Public Health of Spain (CIBERESP), 28029 Madrid, Spain; 11 Teaching and Research in Family Medicine SEMERGEN-UGR, University of Granada, 18016 Granada, Spain

**Keywords:** OXA-48, carbapenem, carbapenemase, Spain, outbreak, multiresistant, enterobacteria, antibiotic stewardship

## Abstract

Carbapenemase-producing Enterobacterales (CPE) are significant contributors to the global public health threat of antimicrobial resistance. OXA-48-like enzymes and their variants are unique carbapenemases with low or null hydrolytic activity toward carbapenems but no intrinsic activity against expanded-spectrum cephalosporins. CPEs have been classified by the WHO as high-priority pathogens given their association with morbidity and mortality and the scarce number of effective antibiotic treatments. In Spain, the frequency of OXA-48 CPE outbreaks is higher than in other European countries, representing the major resistance mechanism of CPEs. Horizontal transfer of plasmids and poor effective antibiotic treatment are additional threats to the correct prevention and control of these hospital outbreaks. One of the most important risk factors is antibiotic pressure, specifically carbapenem overuse. We explored the use of these antibiotics in Spain and analyzed the frequency, characteristics and prevention of CPE outbreaks. Future antibiotic stewardship programs along with specific preventive measures in hospitalized patients must be reinforced and updated in Spain.

## 1. Introduction

Enterobacteriaceae were identified as a public health threat since the discovery of their ability to acquire molecular resistance through extended-spectrum β-lactamases (ESBLs) [1]. In 2016, large-scale genomic sequencing data led to reclassification of various species, originally included in the family Enterobacteriaceae, in an order called Enterobacterales [2]. 

In order to counteract the menace of antibiotic resistant Enterobacterales, carbapenems were developed and introduced into the therapeutic arsenal during the decade of 1990 [3]. Since then, these drugs have been widely used as first-line empirical antibiotic treatment [4]. Nevertheless, this strategy resulted in an even greater problem since the lack in carbapenem stewardship has led to the development of carbapenem-resistant Enterobacterales (CRE) [5,6]. Specifically, the first carbapenemase (NmcA) producer was identified in 1993 in a clinical isolate of *Enterobacter cloacae* [7]. Since then, numerous CRE have been reported [8].

CRE present three main mechanisms of carbapenem resistance: enzyme production, efflux pumps and porin mutations [9]. Of these, enzyme production is the most frequent resistance mechanism and OXA-48-like enzymes represent one of the most common CRE enzymes worldwide [10]. The family takes its name from the first identified enzyme, OXA-48, and includes several sequence variants transmissible via plasmids.

The aim of this review is to analyze the frequency, characteristics and prevention of OXA-48 CRE outbreaks in Spain.

## 2. OXA-48-Like Enzymes: Mechanism of Resistance

**Figure 1:**
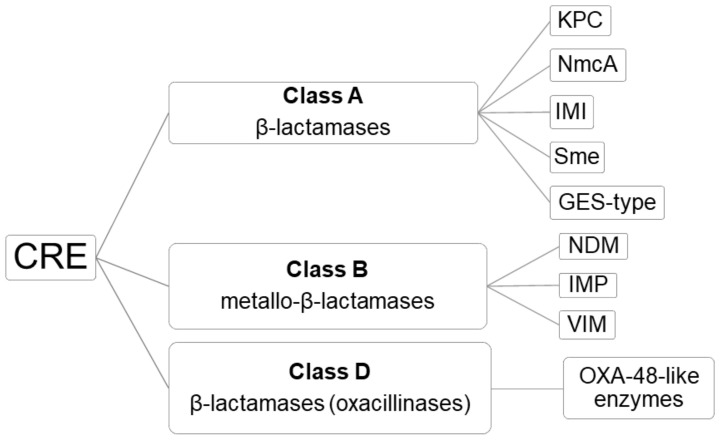
Classification of carbapenemases produced by carbapenem-resistant Enterobacterales (CRE), based on the Ambler classification.

According to the Ambler classification, three classes of carbapenemases can be distinguished in CRE: A, B and D (Figure 1) [11]. According to the Bush–Jacoby functional system [12], carbapenemases in class A include β -lactamases, which are inhibited by clavulanic or boronic acid; class B include metallo- β -lactamases capable of hydrolyzing all ß-lactam antibiotics except aztreonam and inhibited by EDTA and dipicolinic acid; and class D include β-lactamases (oxacillinases) including all OXA-48-like enzymes (e.g., OXA-48, OXA-72 and OXA-244), capable of hydrolyzing carbapenems but not (or weakly hydrolyzing) cephalosporins, and not inhibited by classical inhibitors [13,14,15]. However, there are several OXA-48-like variants, such as OXA-163, that completely lose their ability to hydrolyze carbapenems and display an ESBL phenotype.

The kinetics of OXA-48-like enzymes shows high-level hydrolytic activity against penicillins and low hydrolytic activity toward imipenem and meropenem compared to ertapenem, which is the best substrate for these enzymes [16,17]. Several variants of OXA-48 have been reported in clinical samples since its first identification (Table 1) [15,18,19,20,21,22,23,24,25,26,27,28,29,30,31,32,33,34,35,36,37,38]. Nevertheless, 96 different OXA-48-like enzymes have been reported to date according to the Beta-Lactamase DataBase [39], 35 of which have a definite assignment name by the National Center for Biotechnology Information (NCBI), as shown in the Supplementary Materials (Table S1) [18,19,20,21,22,23,24,25,26,27,28,29,30,31,32,33,34,35,36,37,38,39].

It has been suggested that the progenitor gene of OXA-48 was encoded in *Shewanella* spp., a waterborne bacterium. To date, more than fifteen OXA-48-like variants have been identified in clinical samples, but classical OXA-48 remains as the most frequent form globally. The emergence of this enzyme is mediated by the rapid spread of a broad host-range conjugative plasmid sheltering the *bla*_OXA-48_ gene, located within a composite transposon (Tn*1999*), which flanks the carbapenemase gene and helps mobilize an intervening DNA segment [40,41]. Additionally, five variants of Tn*1999* have been identified to date (Tn*1999.1*, Tn*1999.2*, Tn*1999.3*, Tn*1999.4* and Tn*1999.5*) [41,42,43]. Given the wide transmission and spread of this plasmid, the range of species in which OXA-48 has been identified as part of hospital outbreaks is increasingly higher (Table 2) [44,45,46,47,48,49,50,51,52,53,54,55,56]. This fact led to suggest that transmission of hospital outbreaks might be explained not only by the nosocomial spread of bacteria but also by the horizontal transfer of plasmids between species in certain hospital areas [57]. Apart from human clinical samples, a wide range of OXA-48-like enzymes have been detected in animals or the water environment [39].

*Klebsiella pneumoniae* was the first OXA-48 CRE described, and remains the most common global bacterium related to healthcare-associated infections [46,47], followed by *Enterobacter* spp.

An important aspect that may cause delay in the detection and treatment errors is the attenuated carbapenem hydrolyzing ability of OXA-48 CRE. Given that these bacteria present lower carbapenem hydrolyzing capacity than Ambler type A or B CRE, they sometimes do not exceed the level points for the detection of phenotypic resistance. Therefore, without genotypic diagnostic tools (e.g., polymerase chain reaction (PCR)), OXA-48 CRE may go unnoticed and appear susceptible to carbapenems. However, patients do not show improvement after treatment with carbapenems. This could be explained by the sum of low-level resistance mechanisms and selection of especially resistant subpopulations favored by antibiotic pressure [58].

### Detection of OXA-48 CRE

OXA-48 CRE is of major concern due to the difficulties in their detection and their association with treatment failure. These bacteria might be detected through screening tests, phenotypic detection assays and molecular-based detection methods [48]. The main phenotypic assays include inhibitor-based synergy tests, such as the double-disk synergy test (DDST) or combined disk test (CDT); the Carba NP test; colorimetric tests like the β-CARBA test (Bio-Rad, Marne la Coquette, France); the carbapenem inactivation method (CIM) in water containing a meropenem disk; indirect carbapenemase tests in paper test devices; the lateral flow immunoassays like the OXA-48 K-SeT assay (Coris Bioconcept, Gembloux, Belgium), which have been upgraded to detect multiple enzymes (e.g., KPC, NDM or VIM); electrochemical assays like the BYG Carba test; spectrophotometry and mass spectrometry like matrix-assisted laser desorption ionization–time of flight mass spectrometry (MALDI-TOF MS)-based methods.

The genotypic molecular-based detection methods include PCR, in-house multiplex PCR assays for detecting different carbapenemase genes, commercial assays like microarray-based assays, fully automated systems based on PCR or microarray techniques and whole-genome sequencing based on next generation sequencing technology.

A comprehensive description of all the different detection methods for OXA-48 CRE can be consulted in the recent review by Pitout et al. [48].

## 3. Worldwide Spread

In the last years, surveillance studies have pointed that OXA-48-like cabarpenemases are the most common carbapenemases in several areas of the world [10,15,16,17]. Moreover, they are increasingly being introduced into non-endemic regions where they cause nosocomial outbreaks [48].

A search in PubMed using the terms “OXA-48” and “outbreak” reveals an increasing number of reports in the last years, from 3 in 2010 to 76 in 2020.

The first identification of this enzyme was reported in *Klebsiella pneumoniae* isolated from a urinary tract infection sample in 2001 in Turkey [18]. Since then, a number of nosocomial outbreaks of these bacteria have been reported in this country [59,60]. A rapid spread led to first-identification reports in colonization or infection samples in many areas of the world, especially the Mediterranean region [61].

In Europe, the first case of OXA-48 identification was reported in Belgium in 2008 [62]. In Africa, reports of carbapenem-hydrolyzing OXA-48 β-lactamase in *K. pneumoniae* were communicated in Tunisia in 2010 [63]. In Asia, reports of OXA-48 presence were published in 2012 in Kuwait [64]. In North America, it was first described in the United States in 2013 [65], whereas in South America, the first identification was reported in Brazil in 2014 [29]. In total, more than 50 countries have reported outbreaks of OXA-48-producing bacteria to date [48,66]. In Spain, reports of OXA-48-producing bacteria in outbreaks were published in 2013 [26,27,67]. Since then, several outbreaks have been identified and OXA-48-producing bacteria have become one of the major causal agents of hospital outbreaks in our country [10,42,50].

## 4. OXA-48 CRE Outbreaks in Spain

### 4.1. Frequency

According to the report from Spain on the prevalence of healthcare-related and community infections and the use of antibiotics (EPINE-EPPS study) [68], the prevalence of CRE colonization or infections in Spanish hospital settings is continuously increasing. This study analyzed the prevalence of healthcare-associated infections in one month of the year throughout several hospitals across all the national territory. The prevalence of CRE in Spanish hospitals in 2019 is shown in Table 3. Data from 2020 are not available because the study was stopped due to the COVID-19 pandemic, which especially affected healthcare professionals [69] and hospitalized patients [70] in Spain.

It can be seen that the prevalence of CRE is high in Spanish hospitals, especially regarding nosocomial infections. The bacteria with a higher percentage of carbapenem resistance were *Klebsiella* spp., *Citrobacter* spp. and *Enterobacter* spp. Overall, OXA-48 represents the main enzymatic resistance mechanism, especially regarding *Klebsiella* spp. and *Enterobacter* spp. strains. Recent studies pointed *Acinetobacter baumannii* as a producer of different OXA carbapenemase encoding genes such as OXA-24-like genes [71]. However, as *Pseudomonas* spp. and *Acinetobacter* spp. are not *Enterobacterales*, we did not include them in the present review.

According to a multicenter study performed in 83 Spanish hospitals, OXA-48 was the most frequent carbapenemase produced by CRE (71.5%), followed by VIM-1 (25.3%) [72].

In the study by Hernández-García et al. [73], the incidence of colonization by CRE in a Spanish hospital in Madrid was 2% (161/8209), and more than 50% of them acquired colonization after admission. The main colonizing pathogen was *K. pneumoniae* (54%), followed by *Escherichia coli* (19%). The main resistance mechanism was the expression of OXA-48 (64%) and VIM-1 enzymes (27%), identified through PCR.

A recent study, also performed in Madrid [74], showed that the intestinal loads of OXA-48 colonized patients were high, suggesting an increasing replacement of the host microbiota by OXA-48-producing *K. pneumoniae*. The main factors associated with these loads were the previous antibiotic treatment and development of infection instead of colonization. Another study by Mateos et al. [75] also reported high frequency of *Enterobacter* spp. producing carbapenemase in hospitalized patients, with superimposition of OXA-48 over VIM producers in the last years.

In a previous study by our research group, we identified elderly patients living in residential care homes as a particularly vulnerable population for colonization by OXA-48 CRE [76]. Specifically, 34.5% of these patients showed positive rectal colonization by multiresistant bacteria, 27.5% of them by OXA-48-producing *K. pneumoniae* and 2.5% by OXA-48-producing *E. coli*.

According to the EUSCAPE study [77], a multinational study performed across different European countries, the rate of infections by carbapenemase-producing *K. pneumoniae* or *E. coli* in Spain was 4.01 per 10,000 admissions, one of the highest rates of all the 36 countries analyzed and far over the average rate (1.3/10,000). The ratio between *K. pneumoniae* and *E. coli* carbapenemase producers was approximately 7:1. Among them, OXA-48-like enzymes represented the major resistance mechanism (25.8% in Europe and 69.8% in Spain, the highest after Turkey, 79.0%, and Romania, 73.5%).

Therefore, Spanish hospitals present an alarming increase in OXA-48-producing CRE outbreaks compared to the rest of the European countries.

### 4.2. Surgical Site Infections

The hospital outbreaks caused by OXA-48 CRE do not only occur in hospitalization rooms, but also in surgical patients. A recent study performed by Mora-Guzmán et al. [78] on 65 OXA-48 infected patients showed a significantly increased risk of intra-abdominal OXA-48 CRE infection after surgery following a broad-spectrum antibiotic consumption. The authors advocated a more targeted antibiotic approach to reduce this risk.

The Spanish national program called “Zero Surgical Infection” [79] was designed to reduce the risk of surgical site infections through the implementation of preventive measures such as:

1. Adequate antibiotic prophylaxis before surgery.
2. Skin antisepsis with 2% alcoholic chlorhexidine.
3. Correct hair removal when necessary, without causing injuries or irritation.
4. Adequate control of temperature and glycemia during the procedure.
5. Rapid identification of infection signs after the surgery.

Although few studies have specifically addressed the risk of CRE infection after surgical procedures [78,80], antibiotic pressure and long hospital stay have been reported to be the most important risk factors.

### 4.3. Colonization in the Intensive Care Unit (ICU)

There is a high prevalence of CRE isolates from clinical samples in ICU admitted patients in Spain. In a recent multicenter study performed in eight hospitals, 20% of all carbapenemase-producing *K. pneumoniae* infections were found in ICU patients [81].

A recent prospective cohort study reported that *K. pneumoniae* was the predominant CRE in a Spanish ICU (73.4%), with OXA-48 being the major resistant mechanism [82]. 

These alarming findings led the Spanish government to implement a program called “Zero-Resistance Program” aimed at rapid identification of these patients in order to perform the adequate isolation and prevention measures [83]. The program consists of performing a systematic rectal swab test in all ICU patients at admission and every 48–72 h and applying special preventive measures in CRE colonized patients.

Risk factors for OXA-48 CRE colonization in a Spanish ICU were recently analyzed by Maseda et al. [84]. In their study, OXA-48 was the main carbapenemase found (76.1%) and carriers of CRE showed increased rates of previous chronic diseases, previous digestive or biliary endoscopy, previous hospitalization, ICU admission, intra-abdominal surgery and higher (i.e., worst) clinical prognostic scores. They also presented higher rates of antibiotic intake according to multivariate analyses, especially third or fourth generation cephalosporins (OR = 27.96, 95%CI = 6.88, 113.58, *p* < 0.001) and β-lactam/β-lactamase inhibitors (OR = 11.71, 95%CI = 4.51, 30.43, *p* < 0.001) [85]. This study may provide relevant clues for developing prevention measures to reduce CRE carriers in Spanish ICUs.

In fact, effective preventive measures in the ICU have been reported. For example, successful control of two simultaneous CREs including OXA-48-producing Enterobacterales was described in a hospital in Madrid [85]. These measures included isolation of affected patients in individual confined areas with dedicated personnel to take care of them, 2% chlorhexidine soap for patient daily hygiene, contact precautions including correct hand hygiene (detailed in [86], according to each type of microorganism), in-depth cleaning of the ICU with chlorine solution and vaporized hydrogen peroxide and strict surveillance of environmental samples [85]. Therefore, environmental hygiene might play a crucial role in controlling OXA-48 CRE outbreaks in Spanish ICUs.

### 4.4. Plasmid Transfer

The epidemiology of OXA-48 CRE outbreaks is very complex because, in addition to the transmission of bacteria, horizontal transfer of plasmids is possible.

Some high-risk *K. pneumoniae* clones, like ST307 and ST147, acquired plasmids with various carbapenemases including OXA-48 in several European countries during the late 2000s [87]. Since then, plasmid transfer has become an important factor to explain the rapid spread of this CREs and even the transmission during hospital outbreaks. Therefore, colonization by ESBL-producing *K. pneumoniae* predisposes to future colonization by OXA-48-producing *K. pneumoniae* [84]. This mechanism of transmission must be considered when adopting preventive measures to control hospital outbreaks. During the year 2019, an outbreak in the Netherlands involving 148 OXA-48 CRE colonized patients suggested a horizontal transmission mechanism of this resistance gene by one or more plasmids [88]. Specifically, *bla*_OXA-48_ plasmids were identified in transposon Tn*1999.2* and located on a ca 62 kb IncL/M conjugative plasmid in 14 different species. Accordingly, a ca 62 kb plasmid was responsible for the OXA-48 outbreak [89]. These outbreaks provide strong evidence of both within-host interspecies and between-host dissemination of plasmid-based OXA-48 during a nosocomial outbreak. In Iran, *K. pneumoniae bla*_OXA-48_ plasmids were successfully transferred to an *E. coli* K12-recipient strain, which highlights the importance of horizontal gene transfer in the dissemination of these genes [89]. However, most reported plasmids are conjugative but absent in multiple countries or species, suggesting limited interspecies and interboundary transmission of a common plasmid [90]. In this regard, factors of incompatibility of similar plasmids might be considered, as firstly described in 1973 [91].

Clearly, the transfer of resistance plasmids calls for improved infection control measures to prevent further spread of multiresistant pathogens in future hospital outbreaks in Spain.

### 4.5. Risk Factors for Acquiring OXA-48 CREs

There are limited data regarding the main preventable risk factors for carrying or acquiring OXA-48 CREs. The main reservoir is the gastrointestinal tract [15], thus rectal swab tests represent an adequate sample for their identification [48,92]. As discussed above, admission to ICU is a risk factor for acquiring these strains, probably due to several reasons, such as long-term ventilator use [93], antibiotic pressure or presence of CREs or plasmids in the ICU area. A recent case-control study performed in Korea [94] showed that pneumonia/chronic pulmonary disease, previous fluoroquinolone use and previous use of nasogastric tube were the significant risk factors for CRE infection or colonization in ICU-admitted patients, although no OXA-48-like CRE were identified in their study.

Use of antacids [95] and traveling to endemic countries [96] are also possible risk factors [15]. Finally, vulnerable conditions such as current chemotherapy use or dialysis in the previous 12 months and history of an overnight stay in a healthcare setting or epidemiological linkage to a known carrier also increase the risk for carriage of CRE [97]. However, the main factor associated with new colonization and transmission of CREs is, undoubtedly, antibiotic treatment [95], especially inappropriate carbapenem and aminoglycoside stewardship [93].

In Spain, overuse of carbapenems in hospital settings has been widely reported [98,99].

An overall increase in the use of carbapenems in acute care hospitals has been reported (88.4% higher from 2008 to 2015). They are mainly used empirically (76.2%) for the treatment of urinary tract and intra-abdominal infections in the context of suspicion of polymicrobial or mixed infection (27.4%) and severity (25.4%) [97]. The national Program for Optimizing the Use of Antibiotics (PROA) [100] aims to adequately control the use of antibiotics in our country. For instance, to avoid overuse of carbapenems, other antibiotics have been proposed as an alternative for the treatment of ESBL-producing Enterobacterales [99,101]. The massive consumption of antibiotics, especially in the ICU, calls for a deep reflection and implementation of antibiotic stewardship programs, as highlighted by Timsit et al. [102]. The worrying increase in carbapenem use found in other Spanish hospitals led us to investigate the rate of consumption of each antibiotic in our context. Accordingly, to add further information to the recent studies that have been previously mentioned, we analyzed the original data regarding antibiotic stewardship in hospitalized patients in Spain, available at the EPINE-EPPS Study [68] (Table 4). We included the most used antibiotics in Spain. However, prevalence of other commonly used antibiotics like erythromycin (0.6%), cefepime (0.6%), teicoplanin (0.6%), rifampicin (0.4%) or colistin (0.3%), among others, should be also taken into account.

As shown in the table, carbapenems are the fourth most frequently used group of antibiotics in Spanish hospitals (7%), with meropenem being the most common (5.7%), followed by ertapenem (1.2%) and the combination of imipenem and cilastatin (1%).

**Figure 2:**
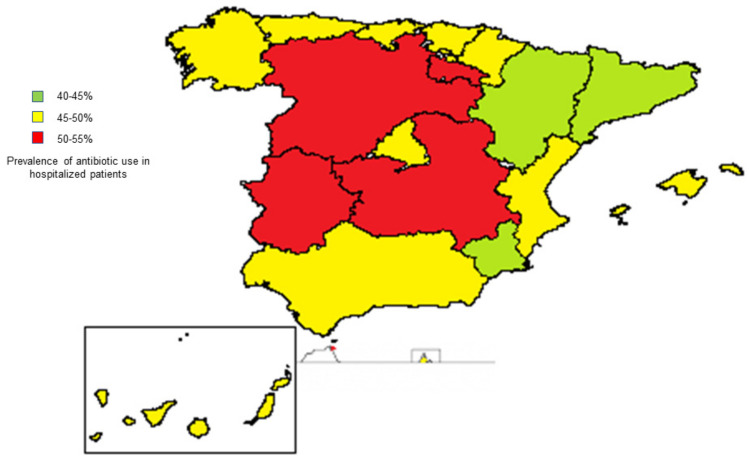
Prevalence of antibiotic use in hospitalized patients in the different autonomous communities in Spain in 2019. Ceuta (red) and Melilla (yellow) are located in North Africa. The percentages represent the prevalence of antibiotic use in hospitalized patients registered in the report from Spain on the prevalence of healthcare-related and community infections and the use of antibiotics (EPINE-EPPS study) in the year 2019 [68].

The overall use of antibiotics in hospitalized patients in Spain is approximately 50% [68]. However, there are some regional differences (Figure 2), ranging from 40.5% in Catalonia to 57.1% in Ceuta.

These data reinforce the need for developing stewardship interventions in Spain in order to homogenize antibiotic use in hospitalized patients and prevent carbapenem overuse to reduce CRE outbreaks.

To achieve this, all the healthcare professionals should be involved in these interventions. At the community level, apart from primary care professionals, pharmacists have been suggested to play an important role in antibiotic use programs [103]. At the hospital level, professionals from all the services, not only ICU, infectious diseases or surgical settings should be aware and conscious of antibiotic stewardship programs and interhospital or interservices sessions should be continuously reinforced and updated. In this regard, infectious diseases, microbiology and preventive medicine and public health services should join forces to design these strategies for ensuring the adequate use of antibiotics and controlling multiresistant outbreaks in Spanish hospitals.

## 5. Treatment

Optimizing therapy for CRE infections represents a fundamental and increasing research field, continuously updating and adapting to the epidemiological contexts. 

The OXA-48 enzyme hydrolyzes carbapenems but shows very weak activity against extended-spectrum cephalosporins such as cefepime and ceftazidime [15], although isolates are frequently multidrug resistant as they combine multiple resistance mechanisms [104].

CRE have historically been susceptible to polymyxins, tigecycline or aminoglycosides (especially gentamicin). Accordingly, these treatments have been widely used as antibiotics of choice for CRE infections. However, varying rates of resistance to all of them have been reported, thus different antibiotics are continuously being tested. Treatment of OXA-48 CRE with ceftazidime-avibactam could be effective according to a study conducted in Italy [105], which is consistent with the resistance profile of an OXA-48 CRE outbreak in Russia [106]. A study performed in Australia also reported effectiveness of other avibactam combinations (imipenem-avibactam or aztreonam-avibactam), suggesting that avibactam may be the most potent β-lactamase inhibitor [107]. Although the authors stated that combined therapy is more effective, successful outcomes were observed in 70% of the patients treated with ceftazidime-avibactam in monotherapy. Carbapenem in combination with amikacin or colistin may be useful in certain cases, but recent reports of resistance are concerning [106].

In Spain, treatment for CRE with ceftazidime-avibactam has proven to be promising, and other novel combinations including meropenem-vaborbactam, imipenem-relebactam, plazomicin, cefiderocol, eravacycline and aztreonam-avibactam [108]. However, meropenem-vaborbactam [109] and imipenem-relebactam [110] are not effective against OXA-48 producers, and limited data are available on eravacycline against CRE [111]. Therefore, the β-lactamase inhibitor with more contrasted efficacy on OXA-48 producers is avibactam. Regarding cephalosporins, despite the paucity of clinical data and the fact that OXA-48 CRE are associated with ESLBs (which implies resistance to cephalosporins), a recent Spanish systematic review suggested that ceftazidime without avibactam could be a therapeutic option [101]. Plazomicin is another therapeutic alternative, which has been reported to be effective in some CREs [112], although it is not commercialized in Spain. Cefiderocol showed encouraging results in terms of efficacy and safety in a clinical trial [113], covering a very wide spectrum of bacteria. Given its siderophore mechanism of action, based on the use of ferric iron transporter systems [114], cefiderocol has been referred to as a “Trojan horse” antibiotic. This drug is available in Spain and has been advocated as a promising future therapeutic option. Finally, ertapenem and meropenem have shown in vitro synergistic activity against CRE [115], although they are not currently recommended for the treatment of OXA-48 producers. Other new drugs with activity against some CPE isolates are at different stages of development [101] and will probably be incorporated into the commercialized therapeutic arsenal in Spain in the next years.

It is important to note that several OXA-48-like variants, such as OXA-163, completely lose their ability to hydrolyze carbapenems and display an ESBL phenotype. Accordingly, they hydrolyze ceftazidime and confer high levels of resistance to this drug when expressed in Enterobacterales. 

Overall, the therapeutic approach to OXA-48 CRE infections must be individualized according to susceptibility, type and severity of infection, and to the characteristics of the patient. Limited data are currently available on the best strategy for OXA-48 CRE, thus future studies might be crucial for optimizing the therapeutic approaches.

## 6. Conclusions

The frequency of CRE colonization or infection is increasing in hospital outbreaks around the world. In Spain, this frequency is higher than the European average. OXA-48 is the most common enzymatic resistance mechanism of CRE in Spain [116]. Colonization in the ICU and infections of the surgical site are especially complicated and, therefore, specific preventive measures should be implemented in these settings. Plasmid transfer between different species has been described, and the presence of possible host bacteria in patients should be considered in order to implement appropriate isolation and preventive measures when an OXA-48 outbreak is identified. One of the most preventable risk factors for hospital outbreaks caused by OXA-48 bacteria is a lack of adequate antibiotic stewardship. In Spain, an overall high frequency of carbapenem overuse (especially ertapenem) has been described in 2019, although regional differences in the percentage of antibiotic use in hospitalization were also reported. Accordingly, specific programs focused on optimizing antibiotic stewardship are required. Finally, preventive control measures need to be reinforced and continuously updated and adapted to identify and control OXA-48 CRE in Spanish hospitals with the aim to stop this growing global health threat.

## Figures and Tables

**Table 1:** OXA-48-like enzymes reported in human clinical samples.

Enzyme	First Clinical Identification	Reference
OXA-48	Turkey, 2001	[18]
OXA-54	France, 2003	[19]
OXA-162	Turkey, 2012	[20]
OXA-163	Argentina, 2008	[21]
OXA-181	India, 2010	[22]
OXA-199	China, 2012	[23]
OXA-204	Tunisia/France, 2012	[24]
OXA-232	India/France, 2011	[25]
OXA-244	Spain, 2012	[26]
OXA-245	Spain, 2012	[26]
OXA-247	Argentina, 2010	[27]
OXA-252	Canada, 2014	[28]
OXA-370	Brazil, 2013	[29]
OXA-405	France, 2014	[30]
OXA-416	Italy, 2013	[31]
OXA-436	Denmark, 2015	[32]
OXA-438	Argentina, 2020	[33]
OXA-484	United Kingdom, 2015	[34]
OXA-505	USA, 2018	[35]
OXA-519	Belgium, 2015	[36]
OXA-535	France, 2018	[37]
OXA-566	New Zealand, 2017	[38]

Updated from the information provided by Mairi et al. [15] and the Beta-Lactamase Database [38] available at: http://www.bldb.eu/BLDB.php?prot=D#OXA.

**Table 2:** OXA-48-like enzymes detected in different Enterobacterales species in clinical samples.

First Clinical Identification	References
*Klebsiella pneumoniae*	[44,45,46,47,48]
*Klebsiella oxytoca*	[44,45,48,49]
*Kluyvera* spp.	[48,50]
*Escherichia coli*	[44,45,48,51]
*Proteus mirabilis*	[44,45,48,52]
*Serratia marcescens*	[53]
*Enterobacter cloacae*	[44,45,48,54]
*Enterobacter aerogenes*	[44,45,48]
*Enterobacter sakasakii*	[15]
*Citrobacter freundii*	[44,45,48,55]
*Citrobacter koseri*	[44,45,48]
*Citrobacter braakii*	[15]
*Salmonella enterica*	[44,45,48,56]
*Morganella morganii*	[44,45,48]
*Providencia rettgeri*	[15]
*Raoultella planticola*	[15]

Updated from the information provided by Suay-García et al. [10].

**Table 3:** Prevalence of CRE in hospital settings in 2019 in Spain.

CRE Species	Nosocomial Infections			Community Infections		
	IM	CR-IM	%CR	IM	CR-IM	%CR
*Escherichia coli*	607	10	2.1	1341	12	1.1
*Klebsiella pneumoniae*	316	30	11.2	305	30	11.5
*Klebsiella oxytoca*	60	2	4.1	79	3	5.0
*Klebsiella* spp., other	12	1	10.0	15	0	0.0
*Enterobacter aerogenes*	45	3	8.3	23	0	0.0
*Enterobacter cloacae*	126	9	8.3	92	2	2.3
*Enterobacter* spp., other	17	1	7.7	15	1	12.5
*Citrobacter* spp.	58	4	10.8	49	0	0.0
*Proteus* spp.	127	3	3.4	218	4	2.4
*Serratia marcescens*	66	5	9.6	58	1	2.1
*Serratia* spp., other	3	0	0.0	6	0	0.0
*Morganella* spp.	53	6	12.8	58	0	0.0
Other Enterobacterales	3	0	0.0	8	0	0.0

IM, number of isolated microorganisms; MR-IM, number of carbapenem-resistant microorganisms; %CR, percentage of carbapenem resistance.

**Table 4:** Prevalence of the most frequent antibiotics used in hospitalized patients in 2019 in Spain.

**Antibiotic Group**	**%**
Penicillins including β-lactamase inhibitors	27.5
Fluoroquinolones	15.4
Third generation cephalosporins	12.4
Carbapenems	7.0
Macrolides	3.1
First generation cephalosporins	2.3
**Antibiotic**	**%**
Amoxicillin and clavulanic acid	14.4
Ceftriaxone	9.8
Piperacillin and tazobactam	8.5
Levofloxacin	7.8
Cefazolin	7.4
Meropenem	5.7
Ciprofloxacin	4.7
Linezolid	2.8
Cotrimoxazole	2.1
Vancomycin	2.0
Metronidazole	2.0
Clindamycin	1.9
Cefuroxime	1.8
Azithromycin	1.7
Gentamycin	1.7
Ampicillin	1.4
Cefotaxime	1.2
Ertapenem	1.2
Daptomycin	1.1
Ceftazidime	1.0
Imipenem and cilastatin	1.0
Cloxacillin	0.9
Amikacin	0.9
Amoxicillin	0.8
Fosfomycin	0.6

The table was designed based on the information provided by the report from Spain on the prevalence of healthcare-related and community infections and the use of antibiotics (EPINE-EPPS Study) [68].

## Data Availability

Not applicable.

## Supplementary Materials

The following are available online at https://www.mdpi.com/2079-6382/10/1/89/s1, Table S1: OXA-48-like enzymes reported to date.
