# Supplementary material for: OXA-48 Carbapenemase-Producing Enterobacterales in Spanish Hospitals: An Updated Comprehensive Review on a Rising Antimicrobial Resistance
Source: Antibiotics (Basel). 2021 Jan 18;10(1):89. doi: 10.3390/antibiotics10010089 (PMC7832331; doi:10.3390/antibiotics10010089)
Supplement: Supplementary file 1 [file antibiotics-10-00089-s001.pdf]

Table S1. OXA-48-like enzymes reported to date.

Enzyme	First identification	Reference
OXA-48	Turkey, 2001	[17]
OXA-54	France, 2003	[18]
OXA-162	Turkey, 2012	[19]
OXA-163	Argentina, 2008	[20]
OXA-181	India/Oman, 2010	[21]
OXA-199	China, 2012	[22]
OXA-204	Tunisia/France, 2012	[23]
OXA-232	India/France, 2011	[24]
OXA-244	Spain, 2012	[25]
OXA-245	Spain, 2012	[25]
OXA-247	Argentina, 2010	[26]
OXA-252	Canada, 2014	[27]
OXA-370	Brazil, 2013	[28]
OXA-405	France, 2014	[29]
OXA-416	Italy, 2013	[30]
OXA-436	Denmark, 2015	[31]
OXA-438	Argentina, 2020	[32]
OXA-439	Argentina, 2015	[38]
OXA-484	United Kingdom, 2015	[33]
OXA-505	Spain, 2016	[38]
OXA-514	Indonesia, 2016	[38]
OXA-515	Netherlands, 2016	[38]
OXA-517	France, 2016	[38]
OXA-519	Belgium, 2017	[35]
OXA-535	France, 2018	[36]
OXA-538	Algeria, 2016	[38]
OXA-546	Portugal, 2017	[38]
OXA-547	France, 2017	[38]
OXA-566	New Zealand, 2017	[37]
OXA-567	Argentina, 2017	[38]
OXA-731	Japan, 2018	[38]
OXA-788	Argentina, 2019	[38]
OXA-793	France, 2019	[38]
OXA-833	India, 2019	[38]
OXA-894	China, 2019	[38]
OXA-D20*	Indonesia, 2018	[38]
OXA-D22*	Netherlands, 2016	[38]
OXA-D281*	USA, 2006	[38]
OXA-D282*	USA, 2006	[38]
OXA-D283*	USA, 2006	[38]
OXA-D284*	China, 2013	[38]
OXA-D285*	Netherlands, 2016	[38]
OXA-D287*	Portugal, 2014	[38]
OXA-D288*	Portugal, 2014	[38]
OXA-D289*	Portugal, 2014	[38]
OXA-D290*	Portugal, 2014	[38]
OXA-D291*	Portugal, 2014	[38]

OXA-D292*	Portugal, 2014	[38]
OXA-D293*	Portugal, 2014	[38]
OXA-D294*	Portugal, 2014	[38]
OXA-D295*	Portugal, 2014	[38]
OXA-D296*	Portugal, 2014	[38]
OXA-D297*	Portugal, 2014	[38]
OXA-D298*	Portugal, 2014	[38]
OXA-D299*	Portugal, 2014	[38]
OXA-D300*	Portugal, 2014	[38]
OXA-D301*	Portugal, 2014	[38]
OXA-D302*	Portugal, 2014	[38]
OXA-D303*	Portugal, 2014	[38]
OXA-D304*	Portugal, 2014	[38]
OXA-D305*	Portugal, 2014	[38]
OXA-D306*	China, 2014	[38]
OXA-D307*	Portugal, 2014	[38]
OXA-D308*	Portugal, 2014	[38]
OXA-D309*	Portugal, 2014	[38]
OXA-D310*	Portugal, 2014	[38]
OXA-D311*	Portugal, 2014	[38]
OXA-D312*	Portugal, 2014	[38]
OXA-D313*	Portugal, 2014	[38]
OXA-D314*	Portugal, 2014	[38]
OXA-D315*	Portugal, 2014	[38]
OXA-D316*	Portugal, 2014	[38]
OXA-D317*	Netherlands, 2016	[38]
OXA-D318*	Portugal, 2014	[38]
OXA-D319*	Portugal, 2014	[38]
OXA-D320*	Portugal, 2014	[38]
OXA-D322*	Portugal, 2014	[38]
OXA-D323*	Portugal, 2014	[38]
OXA-D324*	Portugal, 2014	[38]
OXA-D326*	Netherlands, 2016	[38]
OXA-D327*	Portugal, 2014	[38]
OXA-D328*	Portugal, 2014	[38]
OXA-D329*	Portugal, 2014	[38]
OXA-D330*	Portugal, 2014	[38]
OXA-D331*	Portugal, 2014	[38]
OXA-D332*	Portugal, 2014	[38]
OXA-D333*	Portugal, 2014	[38]
OXA-D334*	Portugal, 2014	[38]
OXA-D335*	Netherlands, 2016	[38]
OXA-D336*	Portugal, 2014	[38]
OXA-D337*	Portugal, 2014	[38]
OXA-D338*	Portugal, 2014	[38]
OXA-D339*	Portugal, 2014	[38]
OXA-D340*	Portugal, 2014	[38]
OXA-D855*	USA, 2007	[38]
OXA-D856*	USA, 2007	[38]

Adapted from the information provided by Mairi et al. [12] and the Beta-Lactamase Database [38], updated in December 2020 (available online: http://www.bldb.eu/BLDB.php?prot=D#OXA). * Denotes a

temporary name in the database, while waiting for a definitive assignment by the National Center for Biotechnology Information (NCBI).
